# Anti-inflammatory Effects of Traditional Chinese Medicines on Preclinical *in vivo* Models of Brain Ischemia-Reperfusion-Injury: Prospects for Neuroprotective Drug Discovery and Therapy

**DOI:** 10.3389/fphar.2019.00204

**Published:** 2019-03-07

**Authors:** Tangming Peng, Yizhou Jiang, Mohd Farhan, Philip Lazarovici, Ligang Chen, Wenhua Zheng

**Affiliations:** ^1^Center of Reproduction, Development and Aging, Faculty of Health Sciences, University of Macau, Macau, China; ^2^Institute of Translation Medicine, Faculty of Health Sciences, University of Macau, Macau, China; ^3^Department of Neurosurgery, Affiliated Hospital of Southwest Medical University, Luzhou, China; ^4^Neurosurgical Clinical Research Center of Sichuan Province, Luzhou, China; ^5^Faculty of Medicine, School of Pharmacy, The Institute for Drug Research, The Hebrew University of Jerusalem, Jerusalem, Israel

**Keywords:** anti-inflammatory, traditional Chinese medicine, stroke, ischemia and reperfusion injury, traumatic brain injury

## Abstract

Acquired brain ischemia-and reperfusion-injury (IRI), including both Ischemic stroke (IS) and Traumatic Brain injury (TBI), is one of the most common causes of disability and death in adults and represents a major burden in both western and developing countries worldwide. China’s clinical neurological therapeutic experience in the use of traditional Chinese medicines (TCMs), including TCM-derived active compounds, Chinese herbs, TCM formulations and decoction, in brain IRI diseases indicated a trend of significant improvement in patients’ neurological deficits, calling for blind, placebo-controlled and randomized clinical trials with careful meta-analysis evaluation. There are many TCMs in use for brain IRI therapy in China with significant therapeutic effects in preclinical studies using different brain IRI-animal. The basic hypothesis in this field claims that in order to avoid the toxicity and side effects of the complex TCM formulas, individual isolated and identified compounds that exhibited neuroprotective properties could be used as lead compounds for the development of novel drugs. China’s efforts in promoting TCMs have contributed to an explosive growth of the preclinical research dedicated to the isolation and identification of TCM-derived neuroprotective lead compounds. *Tanshinone*, is a typical example of TCM-derived lead compounds conferring neuroprotection toward IRI in animals with brain middle cerebral artery occlusion (MCAO) or TBI models. Recent reports show the significance of the inflammatory response accompanying brain IRI. This response appears to contribute to both primary and secondary ischemic pathology, and therefore anti-inflammatory strategies have become popular by targeting pro-inflammatory and anti-inflammatory cytokines, other inflammatory mediators, reactive oxygen species, nitric oxide, and several transcriptional factors. Here, we review recent selected studies and discuss further considerations for critical reevaluation of the neuroprotection hypothesis of TCMs in IRI therapy. Moreover, we will emphasize several TCM’s mechanisms of action and attempt to address the most promising compounds and the obstacles to be overcome before they will enter the clinic for IRI therapy. We hope that this review will further help in investigations of neuroprotective effects of novel molecular entities isolated from Chinese herbal medicines and will stimulate performance of clinical trials of Chinese herbal medicine-derived drugs in IRI patients.

## Introduction

### Brain IRI Pathology

Stroke is the second leading cause of death all over the world, killing nearly 6.7 million individuals every year. Among of these stroke individuals, more than 80% patients account for cerebral ischemia, which are mainly contributed to the occlusion of a cerebral artery (Jia J.M. et al., 2017). It’s reported that the 5-year recurrence risk ranges from 15 to 40%. Therefore, the number of patients suffering an initial IS will be increased by approximately 30% in 2023 in comparison to 1983 (Wolfe, 2000). TBI is a major burden to hospitals in terms of utilization of intensive care units (Gennai et al., 2015). The mortality from severe TBI is as high as about 35–40% (Beauchamp et al., 2008). Recent estimates indicate that up to 3 million individuals in the United States suffer a TBI annually (Faul and Coronado, 2015) and the World Health Organization states that TBI will be a major health problem in 2020 (Dang et al., 2017). TBI is defined as a mechanical impact to the head that involves brain tissue damage. Its outcome can be mild, moderate or severe and impacts in all ages and can be caused by various reasons, such as car accidents, sport injuries, falls, etc. Post-TBI is based on a combination of primary and secondary damages (Mustafa and Alshboul, 2013). Primary damage is acute and caused by the mechanical and shearing forces applied at the time of impact (Leker and Shohami, 2002), hematomas, and deformation and destruction of brain tissue, including contusions and axonal injury (Stocchetti et al., 2017). Within the focal “core” region, many of the cells, neurons in particular, die from necrosis (Lipton, 1999; Brouns and De Deyn, 2009). However, amplification of the injury process extends beyond the core region leading to a secondary stage of neuronal death (Zheng et al., 2003; Brouns and De Deyn, 2009) to an area coined as “penumbra” (Astrup et al., 1981; Hossmann, 1994). The delayed secondary brain damage evolves over time with activation of multiple molecular and cellular signaling pathways (Stocchetti et al., 2017) and shares mechanisms similar to those occurring in stroke (Leker and Shohami, 2002), In stroke, the initial injury insult occurs within a few minutes and blocks the blood flow to the brain injured focal core, ultimately leading to a significant reduction in oxygen and glucose supply to neurons. At occlusion center, the focal “core,” the vast majority of the cells, especially neurons, die from necrosis making rescue attempts almost impossible. Furthermore, amplification of the injury extends beyond the core region to “penumbra,” leading to a secondary damage. The reason for damage in this particular region stems paradoxically from the restoration of blood circulation (reperfusion) and resupply of oxygen and glucose. This ischemia-reperfusion-injury (IRI) process accelerates neuronal cell death through energy depletion and triggers a variety of post-ischemic responses which exacerbates brain damage and triggers pathological events, such as increase of ROS, induction of calcium overload, disruption of BBB, activation of apo-necrotic cell death, a huge increase in free radicals, excitotoxicity, inflammation with excess production of inflammatory mediators (Lahiani et al., 2018).

### Pharmacological Models to Study Brain-IRI

Ischemia-and reperfusion-injury results from restricted blood flow to part of the brain that causes an irreversibly damaged ischemic core and a surrounding region of viable, yet functionally impaired brain tissue, known as the penumbra (Moretti et al., 2015). Tissue injury and/or death occur because of the oxygen (hypoxia, anoxia) and glucose (hypoglycemia) deprivation, which is determined primarily by the magnitude and duration of the interruption in the blood supply, and then followed injury resulted from reperfusion (reoxygenation). Although oxygen levels are restored upon reperfusion, a surge in the generation of ROS occurs which exacerbate ischemic injury (Shi and Liu, 2007). During ischemia, ATP levels and intracellular pH decrease, because of anaerobic metabolism and lactate accumulation. As a consequence, ATP-dependent ion transport mechanisms become dysfunctional, resulting in increased intracellular and mitochondrial calcium overload, cell swelling and disintegration, and cell death by different or combined biochemical mechanisms such as necrosis, apo-necrosis, apoptosis, and autophagy (Kalogeris et al., 2012). For assessing potential neuroprotective modalities, the use of well-established cellular, hypoxic/ischemic neuronal model systems *in vitro* is a prerequisite. The majority of the pharmacological *in vitro* models include brain neuronal cultures. These neuronal cultures are deprived of oxygen and glucose, using different protocols and the cell death following these insults is measured (Richard et al., 2010; Holloway and Gavins, 2016). Organotypic brain slices (Noraberg et al., 2005), brain cortical primary neurons (Lazarovici et al., 2012), PC12 cells (Seta et al., 2002; Tabakman et al., 2005) and neuroblastoma cells (Mahesh et al., 2011; Hu et al., 2016) are most commonly used as cellular models *in vitro*, to investigate IRI-induced cell death (neurotoxicity) and for characterization of neuroprotective compounds. These IRI experiments are performed by different experimental paradigms, using tissue culture anaerobic incubators (Zhang et al., 2015), and homemade ischemic devices (Abu Raya et al., 1993; Tabakman et al., 2002), in which the oxygen is replaced by nitrogen or argon gasses to achieve hypoxic or anoxic conditions. These experiments performed with tissue culture media lacking glucose, generate OGD conditions, mimicking the *in vivo* ischemic insult. Re-exposure of these OGD neuronal cultures to atmospheric oxygen conditions for different periods, elicits a reoxygenation insult, simulating the combined hypoxia-hypoglycemic insult achieved in IRI animal models, by obstruction of blood circulation to the brain. Preventing or minimizing the cascade of events causing IRI and cell death, in particular affecting the penumbra would have a more profound effect on the post-ischemic outcome than intervention at later steps in that stroke pathological cascade. This logic is, of course, the motivation of providing the cerebral IRI patient with the earliest possible neuroprotective treatment. Therefore, neuroprotection is defined as any combination of strategies to antagonize, interrupt, or slow down the sequence of noxious biochemical and molecular events, which, if untreated, would lead to irreversible neuronal cell death (Ginsberg, 2008). The increasing biochemical pathways participating in ischemic neuronal cell death (Tuttolomondo et al., 2009) has spurred investigations on a large number of potential neurotherapeutic modalities such as TCMs.

A great number of patients with cerebral IRI express transient or permanent interruption of cerebral blood supply, eventually leading to cerebral infarct (CI). The final CI volume and the neurological deficits depend on a variety of factors such as the duration and severity of ischemia, the presence of a sufficient systemic blood pressure, localization of the CI, and also on age, sex, comorbidities with the respective multi-medication and genetic background. Thus, cerebral IRI is a very complex disease (Sommer, 2017). The most common pharmacological animal models currently in use include *in vivo* focal ischemia models (Durukan and Tatlisumak, 2007) using rodents in which a blood vessel in the brain is occluded. Animal models of stroke and TBI provide an essential tool for the understanding the complex pathophysiology of brain IRI and for testing novel neuroprotective, neuroregenerative, or anti- inflammatory drugs in pre- clinical setting. Brain IRI models can be divided into two categories: (i) models to study how risk factors may contribute to vascular damage that ultimately leads to stroke; (ii) models for the study of the pathophysiological consequences of stroke, and for testing neuroprotection, neuroregeneration or anti- inflammatory effects, including models of focal and global cerebral ischemia. Stroke caused by an acute cerebral vessel occlusion can be reproduced by mechanical occlusion of either the pMCAO (Proximal Middle Cerebral Artery Permanent Occlusion – mainly large vessel occulsion) or distal MCAO (dMCAO) (small vessel occlusion), or bilateral occulsion (Bilateral common carotid artery occlusion (BCCAO), or by thrombotic occlusion (Bacigaluppi et al., 2010). Considering the heterogeneous nature of the clinical manifestation in TBI, numerous TBI models have been established, in particular rodents, according to their small size and standardized measurement results. More-recent models of fluid percussion injury, critical cortical impact injury (CCI), weight drop injury, and blast injury have been employed for improving our understanding of the harmful, complex molecular cascades initiated by TBI (Xiong et al., 2013). Some of these models have been often investigated to establish the neuroprotective role of TCMs.

### Brain IRI-Induced Inflammation

Inflammation contributes significantly to the development of cerebral ischemic pathology. Compared to the rescue of primary damage following IRI, there is a longer therapeutic time window left to secondary damage by brain inflammation. Therefore, anti-inflammation is a favorite therapeutic target (Kawabori and Yenari, 2015). Inflammation is a complex series of interactions between inflammatory cells and molecules, all of which could be either neurotoxic or neuroprotective (Jassam et al., 2017; Malone et al., 2018). Within the first 24 h of brain IRI onset, cerebral IRI is associated with a developing inflammatory response with pathologic contributions from vascular leukocytes and endogenous microglia (Zheng and Yenari, 2004). Signaling chemokines orchestrate the communication between the different inflammatory cell types and the damaged tissue leading to cellular chemotaxis and lesion occupation, especially around the penumbra. That leads to adhesion molecule upregulation in brain and peripheral leukocyte recruitment, respectively. Once activated, inflammatory cells can release numerous cytotoxic agents, such as MMPs, NO and ROS, etc. These changes may cause both cell damage and disruption of the BBB and extracellular matrix (Emsley and Tyrrell, 2002), allowing neurotoxic serum substances to enter the brain, causing edema (Siesjo and Siesjo, 1996). IRI can also activate the SNS and the hypothalamic-pituitary-adrenal axis (HPA) and further stimulate circulating immune cells to enter the brain (Mracsko et al., 2014). These processes are especially pronounced during reperfusion when massive influx of ROS and leukocytes occur into cerebral infarct area. Inhibition of inflammatory cascades has been found to alleviate injury in experimental IRI models (Han and Yenari, 2003). Therefore, the inflammatory cascade response post-cerebral IRI injury contributes to increased damage in early course of ischemic brain injury, and accordingly, targeting anti-inflammatory agents may be a therapeutic strategy in IRI. The brain with IRI responds to the neuronal damage by activating a variety of anti-inflammatory responses to confer neuroprotection, e.g., “repairing” neuronal damage (Yoneyama et al., 2011; Reekmans et al., 2012). Although cytokines released in the brain after ischemia exhibit both pro- and anti-inflammatory effects, the overall balance of the initial post-ischemic inflammatory reaction appears to enhance cell death, neuronal loss, and finally, expansion of the penumbra (Stoll and Jander, 1999). This can lead to neurological and cognitive impairment (Liraz-Zaltsman et al., 2011), in mood, cognition, particularly memory, attention, and executive cognitive functions (Dang et al., 2017), along with motor deficits. Chemoattraction of hematopoietic cells from the blood circulation toward the IRI site includes recruitment of BM-derived mesenchymal (MSC) and hematopoietic stem/progenitor cells (HSC). These cell populations that are involved in the “natural healing” of the damaged brain tissue (Stumm et al., 2002), maintain a balance of pro- and anti-inflammatory cytokines release (Whitney et al., 2009). Endogenous mechanisms of repair and regeneration may be enough for mild damage. However, with severe injuries the endogenous attempts at repairing and restoring neuron activity are mostly slow and incomplete and therefore fail to provide neuroprotection (Madhavan and Collier, 2010; Reekmans et al., 2012). Therefore, endogenous repair alone is not adequate for functional recovery (Reekmans et al., 2012). rtPA is the only agent approved to treat patients with acute IS, while rtPA is harbored with hemorrhage risk and limited to use within 4.5 h of ictus of IRI. Therefore, rtPA is applied in approximately 5% patients with acute IS and there is no drug available for TBI therapy (Gennai et al., 2015; Stocchetti et al., 2017). In recent years, due to the recognition of the complex molecular mechanisms of neuronal cell death following TBI (Kazantsev, 2007), a plethora of agents with neuroprotective effects have been assessed. However, despite much progress, a clinically proven neuroprotective therapy, which is capable of attenuating brain injury and prevent neurodegeneration, is still not found (Bordet et al., 2007). Similarly, a great number of clinical trials investigated the safety and efficacy of different therapeutic strategies for acute IS with unsatisfactory results. These clinical failures have aroused the interest in TCM, which has been used to cure ischemic diseases more than 2000 years. In animal models, a variety of TCMs play a neuroprotective role expressed by alleviation of the ischemic infarction volume, improvement of neurological deficits and attenuation of brain edema through suppressing the activation and/or expression of inflammatory mediators causing inhibition of the inflammatory cascades. In the current review, we briefly describe the inflammatory mediators associated with cerebral IRI and the effect of TCM therapy on these mediators in IRI models and when possible, we addressed the mechanism of TCMs neuroprotective effect in brain IRI.

### Brain IRI and Cytokines

Cytokines are upregulated in the brain post-IRI insults, such as stroke (Kim et al., 2015) and TBI (Aisiku et al., 2016; Zhang M. et al., 2018), and are found not only in cells of the immune system, but discovered in resident brain cells, such as glia and neurons. The most studied cytokines associated with inflammation in cerebral IRI are pro-inflammatory cytokines TNF-α, IL-1, and IL-6, IL-8, IL-10, IL-17 and TGF-β (Lambertsen et al., 2005, 2012). These pro-inflammatory cytokines have been found in patients with acute cerebral ischemia (Nardi et al., 2012), animals with cerebral IRI (Saito et al., 1996) and *in vitro* neuronal models exposed to OGD with or without reoxygenation (Yan et al., 2016). Previous reports demonstrated that pro-inflammatory cytokines are significantly up regulated post-cerebral IRI and different types of cytokines reached peak level from hours to days post-cerebral IRI (Huang et al., 2006; Schwarzmaier et al., 2013). Increased levels of interleukin IL-6, IL-1, IL-8, IL-10, and TNF-α were found significantly related to TBI worse outcomes (Rodney et al., 2018).

#### TNF-α

TNF-α, is massively released in both transient and permanent cerebral IS via up regulation of TNF-α mRNA in brain parenchyma and cortex (Wang et al., 1994) as well as in TBI (Sordillo et al., 2016). The level of TNF-α is increased within 1–3 h following insult and reaches the second peak within 24–36 h (Murakami et al., 2005). TNF-α share some of similarities to IL-1 and IL-6 in the development of inflammatory responses. In MCAO rat model, TNF-α increases significantly, proportionally to IL-1 and IL-6 levels, within hours post-IRI (Liu et al., 1993). Increased levels of TNF-α in CSF and serum was reported to be neurotoxic, increasing infarct volume while decrease in its levels was neuroprotective (Zaremba et al., 2001; Shi et al., 2018; Xu et al., 2018). Kang et al. (2015) reported that in a cohort of 293 patients with IS, the level of expression of TNF-α was increased in the injured cortical side by comparison to the contralateral side and in correlation with the infarct size. TNF-α was initially found in neurons (Liu et al., 1994), then in microglia, astrocytes (Uno et al., 1997) and in peripheral immune system (Offner et al., 2006). TNF-α appears to have pleiotropic functions in IRI (Hallenbeck, 2002). Suppression of TNF-α levels alleviate IRI (Nawashiro et al., 1997b; Hallenbeck, 2002), while administration of recombinant TNF-α protein post-IRI could exacerbate brain injury (Barone et al., 1997). TNF-α appears to be involved in the phenomenon of ischemic tolerance by controlling NF-kappa B trans-activation (Ginis et al., 2002), and in mice deficient in TNF receptors larger brain infarcts have been found (Bruce et al., 1996). TNF-α can activate cytoprotective pathways by pretreatment or continuous exposure at low doses. This property might explain the paradoxical observation that TNF- α improves the survival and function of hypoxic cells and IRI’ mice. The dual effects of TNF-α may be also associated with differential regulation or activation of TNFR1 (p55) and TNFR2 (p75) receptors (Pan and Kastin, 2007). The reasons for this disparity may be related to different pathways through which TNFα signals. Majority of effects induced by TNF-α are exerted by TNFR1, which contains a death domain and may act as a bifurcation point for signaling associated with cell death or survival. Ischemic preconditioning leads to upregulation of neuronal TNFR1 while intracerebral administration of TNFR1 antisense oligodeoxynucleotide, decreased the TNFR1 level, suppressed the ischemic preconditioning-mediated neuroprotective effect, showing that TNFR1 upregulation is implicated in ischemic tolerance (Pradillo et al., 2005). Upregulated expression of TNFR1 mRNA in the ipsilateral cortex was demonstrated during IRI and was significantly increased at 12 h post-reperfusion in MCAO model (Yin et al., 2004). In addition, clinical study also found that TNFR1 levels enhanced in patients with acute stroke (Fassbender et al., 1997). In cerebral IRI, Nawashiro et al. (1997a), found that pre-treatment with TNF-α 48 h before MCAO, induced neuroprotection, while Bruce et al. (1996) found that lack of TNF-α receptors increased the ischemic infarct. The levels of expression of TNF-α is up regulated in IRI, and the IRI mediated-apoptosis is significantly reduced in TNF-R p55-deficient mice (Okazaki et al., 2005). NF-R p75, another TNF-R, may be related with cell survival by activation of transcription factor NF-κB (Plumpe et al., 2000). The binding of TNF-α to TNF-R regulates different proteins, including PKC, tyrosine kinase, MAPK, and PC-PLC (Zhang J. et al., 2018). Of these proteins, MAPK are mostly studied in IRI and is most probably involved in neuroprotection, while c-Jun NH2-terminal kinases and p38 kinases contribute to neurotoxicity. In line with these studies, treatment of MCAO animals with either chimeric monoclonal antibody against TNF-α (Infliximab) or Etanercept, a chimeric fusion protein of TNF-α receptor-2 with a fragment region of Immunoglobulin G (IgG1), conferred neuroprotection to rat brain from focal IRI (Arango-Davila et al., 2015). Similarly, Etanercept, has a significant effect of decreasing microglia activation in experimental TBI models, inducing a limitation of TBI-induced cerebral ischemia, amelioration of brain contusion signs, as well as of the motor and cognitive dysfunctions. On this basis, it appears that TNF-α antagonism may improve outcomes of TBI, although further investigations in clinical trials are needed to confirm these experimental results (Tuttolomondo et al., 2014).

#### Interleukin 1

IL-1 isoforms, IL-1α and IL-1β and its endogenous inhibitor, IL-1 receptor antagonist (IL-1Ra) have been the most studied in experimental cerebral IRI models known to be produced by microglia, whereas IL-1β is also synthesized by neurovascular units (Luheshi et al., 2011). Both isomers act mainly through two receptor types, named as type I and type II. Type I receptor is detected in great number of cells and can bind both isomers, while type II receptor is detected on the cell surface of neutrophils, type B lymphocytes, and macrophages, and only binds IL-1β. Post-cerebral IRI induced in experimental rats, indicate that IL-1β mRNA is firstly increased in 15–30 min, and is responsible for the up regulation of IL-1 protein a few hours later (Brea et al., 2009). Furthermore, IL-1β is observed 2–6 h post-cerebral IR injury and reaches its peak at 12–24 h (Wu et al., 2018). Several studies found a direct correlation between the increase secretion of IL-1 in IRI brain cortex and the worsening of infarct severity while IL-1Ra inhibited neuronal damage and improved the outcome *in vivo* (Allan et al., 2005; Pang et al., 2015). IL-1 β mRNA increase has been recorded within 15–30 min’ post-stroke induction (Buttini et al., 1994) with IL-1 β protein increased within hours (Davies et al., 1998). After 20 min of transient global cerebral stroke in rats, both IL-1 β mRNA and protein expression increase not only during early reperfusion (1 h), but also after a delayed period (6–24 h) (Haqqani et al., 2005). Consistent with the hypothesis of IL-1β induced neurotoxicity, aggravated brain injury occurred when IL-1β was administered to rats (Yamasaki et al., 1995), while IL-1 knockout mice had smaller infarct volume compared to wild type. Overexpression or treatment with IL-1Ra alleviated cerebral infarct area (Mulcahy et al., 2003), while IL-1Ra deficient mice showed an increase of cerebral infarct area in IRI (Pinteaux et al., 2006). Inactivating or knocking out the IL-1R1 decreased the extent of damage caused by IRI inducing neuroprotection (Basu et al., 2005). Similar effects were reported on TBI models (Sun et al., 2017; Chung et al., 2018; Newell et al., 2018).

Many studies indicated that MAPK pathway is activated in cerebral IRI, and increased phosphorylation of extracellular regulated protein kinases Erk1/2 was found already 30 min post-injury in the ischemic core or penumbra (Liu R. et al., 2018). Furthermore, inhibition of the Erk 1/2 caused down regulation of IL-1β expression and neuroprotection, while brain intraventricular injection of IL-1β significantly elevated infarct area, inducing influx of neutrophils and aggravated brain edema (Um et al., 2003). Moreover, it was also found that IL-1 β can induce the expression of endothelial cell adhesion molecules. IL-1β promoted the release of chemokines, up-regulation of VCAM-1 and ICAM (ICAM-1, ICAM-2), increasing the expression of MMP-9, activation of chemokines, contributing to the influx of neutrophils into the injured brain area and contributing to tissue necrosis (Thornton et al., 2008; Hoyte et al., 2010; Allen et al., 2012; Cheng et al., 2012).

#### Interleukin 6

IL-6, like IL-1β, also plays an important role in the IRI-induced pathophysiological cerebral process, leading to secondary damage (Lu et al., 2018). In animals with cerebral IRI, IL-6 mRNA expression increased within 3 h post-MCAO, reached peak levels at 12 h post-brain ischemic damage, and lasted for days (Tarkowski et al., 1995). IL-6 deficient mice have similar cerebral infarct volume compared to wild type, suggesting that IL-6 does not participate in ischemic pathogenesis (Clark et al., 2000). However, other studies suggest either a helpful (Herrmann et al., 2003) or harmful role (Smith et al., 2004). In reports focusing on patients with acute stroke, IL-6 levels in both CSF and blood plasma was increased within a few hours of onset, and the level of IL-6 was proposed as an effective marker of early neurological deterioration. In addition, release of IL-6 was directly related to increased area of brain ischemic infarct and worsening of the clinical outcome (Waje-Andreassen et al., 2005; Huang et al., 2006). Interestingly, some data proposed that IL-6 may be a marker in IRI since treatment with recombinant human IL-6 significantly attenuated the cerebral IRI in MCAO model, conferring neuroprotection (Relton et al., 1996; Loddick et al., 1998; Scheller et al., 2011).

#### Interleukin 8

The chemokine interleukin-8 (IL-8) and its receptor CXCR2, is a pro-inflammatory cytokine, with potent chemotactic and activating properties on neutrophils (Baggiolini et al., 1992). Numerous cells can generate IL-8, such as monocytes/macrophages, endothelial cells, and neutrophils following stimulation with IL-1 and TNF-α (Bazzoni et al., 1991). Astrocytes produce IL-8 in response to IL-1 and TNF-α challenge, whereas constitutive expression of IL-8 is found in activated and transformed astrocytes (Nitta et al., 1992). In addition, IL-8 induced neurotrophic activity on hippocampal neurons (Araujo and Cotman, 1993). IL-8, is secreted by number of cell types, and the mainly cellular sources of IL-8 are monocytes and macrophages (Apostolakis et al., 2009). IL-8 can recruit and promote the activation of monocytes and neutrophils in response to acute inflammation (Goto et al., 2004). Compared to majority of other inflammatory cytokines, the activation of IL-8 in experimental IS (DeForge et al., 1992) and TBI (Helmy et al., 2011) is from hours in early phase and lasts for days or even for weeks. In addition, several reports show that IL-8 has the ability to regulate the antioxidant gene expression, which in turn can down regulate of the production of IL-8 in cerebral IRI, reducing the ischemic infarct area and conferring neuroprotection (Zhang C. et al., 2014).

#### Interleukin 10

IL-10, is an anti-inflammatory cytokine expressed in response to brain damage, where it facilitates the resolution of inflammatory cascades. IL-10 acts through inhibiting IL-1 and TNF-α increase levels, and can suppress TNF-α cytokine receptor expression and reduce receptor activation (Strle et al., 2001). The level of IL-10 increases in experimental models of cerebral IRI and in clinical IS patients (Liang et al., 2015; Lu et al., 2017). Both exogenous administration (Spera et al., 1998) and gene transfer of IL-10 (Ooboshi et al., 2005) in IRI animal models indicated a neuroprotective role. IL-10 expression is enhanced in the early phase of acute IS, and its plasma level represents a risk factor in patients with subcortical infarcts (Vila et al., 2003). Patients with acute stroke have an elevated numbers of peripheral blood mononuclear cells secreting IL-10 (Pelidou et al., 1999) and its concentration is increased in CSF (Tarkowski et al., 1997). Similarly, it was reported that IL-10 plays a neuroprotective effect on preclinical TBI models (Garcia et al., 2017).

#### TGF-β

TGF-β, belonging to the transforming growth factor superfamily, includes several different isoforms: TGF-β1, TGF-β2, and TGF-β3 and is produced by all lymphocyte’s cell lineages. TGF-β binds to the three isoforms of the TGF-β receptor (TGFBR) with different affinities. TGFBR1 and 2 are both serine/threonine and tyrosine kinases, but TGFBR3 does not have any kinase activity. They are necessary for activating canonical or non-canonical signaling pathways, as well as for activation of other signaling pathway which regulate several downstream cellular substrates and regulatory proteins, for example, transcription of different target genes that have role in differentiation, chemotaxis, proliferation, and activation of many immune cells (Vander Ark et al., 2018) TGF-β is recognized as an damage-induced factor, which is strongly up-regulated in many acute or chronic CNS diseases, such as stroke and TBI, and is generally considered to induce neuroprotection (Vivien and Ali, 2006). In cerebral IRI, the expression of TGF-β mRNA is up regulated 1–6 h post-insult and is maintained a high level for up to 15 to 21 days. A direct correlation was found between TGF-β expression and infarct volume or inflammatory response (Pang et al., 2001). Expression of TGF-β in IRI side is higher than contralateral side, and predominant in the penumbra (Krupinski et al., 1996). TGF-β play its neuroprotective effect upon direct injection into the penumbra not in the ischemic core (Johnston et al., 2001). Treatment with TGF-β inhibitor can exacerbate the IRI lesions (Ruocco et al., 1999). TGF-β1 knockdown reduced the number of neurons, inhibited astrogliosis and lead to a significant neurological deficit in rats with TBI. Subsequently, Smad3, a downstream signaling molecule of TGF-β1 receptor, was reported to participate in pericontusional region post-TBI (Wang X.Y. et al., 2015) Cumulatively these evidences support the claim of the neuroprotective role of TGF-β in cerebral IRI. The mechanism of TGF-β induced-neuroprotection may be explained by (1) preservation of cerebral blood flow (McNeill et al., 1994; Liao et al., 2016); (2) inhibition of neutrophil adherence to endothelial cells (Li et al., 2016; Li D. et al., 2018); (3) inhibition of macrophages activation resulting in decreased levels of oxygen-and nitrogen-derived radical species; (4) decrease levels of pro-inflammatory cytokines (Deng et al., 2016; Xiong D. et al., 2016); (5) down-regulation of the calcium channels Cav1.2 in cortical neurons (Liu Z. et al., 2018); (6) activation of down-stream Smad3 signals (Wang X.Y. et al., 2015), etc.

#### Toll-Like Receptors

Toll-like receptor family is one of the main executors of inflammation after interaction with DAMP like HMGB1 or HSP family from damaged cells (Lin et al., 2011). Many studies have indicated the neuroprotective role of TLRs in IS via modulation of neuroinflammation (Gesuete et al., 2014). Extracellular domain of TLRs bind either exogenous pathogens or endogenous DAMP originating from degraded extracellular matrix proteins or intracellular contents released after cellular demise. After binding and activation, TLRs stimulate downstream signaling pathways and recruit a set of adaptors leading to the activation of downstream kinases and nuclear factors which regulate the expression of inflammatory genes, promoting production of inflammatory cytokines in microglia cells (Lehnardt, 2010). TLRs are found in various glial cells, such as microglia, astrocytes, and oligodendrocytes. Both microglia and astrocytes are activated when their TLRs bind to corresponding ligands to induce synthesis and release of proinflammatory cytokines (Jack et al., 2005; Lv et al., 2011; Li M. et al., 2018). In brain IRI injury, the TLRs are activated in neurons and TLR2 and TLR4 participate in the inflammatory response through binding to the endogenous ligands, such as HSP and HMGB1 (Liao et al., 2016; Seong et al., 2018). Hyakkoku et al. (2010) demonstrated that the infarct volume decreased significantly in TLR4 knock-out mice in comparison with wild type mice, while in TLR9 knock-out mice this neuroprotective effect was absent. TLR2 and TLR4 bind to their respective ligands to form dimeric complexes and activate its downstream adaptors, such as MyD88, TIR domain containing adaptor protein (TIRAP/MyD88) adaptor-like (Mal), TIR domain-containing adaptor-inducing IFN-β (TRIF), TRAM, and SARM (O’Neill and Bowie, 2007). In addition, TLR2 and TLR4 in MCAO models induced release of the proinflammatory cytokines, IL-1β and TNF-α, through MyD88-dependent and MyD88-independent pathways. Tang et al. (2007) found that overexpression of TLR4 mRNA within 1 h of transient cerebral ischemia resulted in increasing expression of multiple inflammatory cytokines, such as TNF-α and IL-1β. In addition, other studies indicated that TLR2 knock-out mice can down regulate MCP-1, which is a proinflammatory chemokine (Bohacek et al., 2012; Chen et al., 2015). Cumulative evidences confirm the involvement of TLR2 and TLR4 in cerebral IRI inflammatory processes. However, other studies indicate that TLRs beside regulation of neuroinflammation (van Noort and Bsibsi, 2009) have additional functions such as protection of oligodendrocyte toward ischemic cell death and demyelination in white matter stroke models (Choi et al., 2014, 2017).

#### Brain IRI and Nitric Oxide

Nitric oxide is a well-known endothelium-derived relaxing factor known as a second messenger not only involved in the control of vasomotor tone but also in vascular homeostasis, neuronal, and immunological functions (Berdeaux, 1993). NOS is the enzyme generating NO by the conversion of l-arginine to l-citrulline. NO is generated by the NOS, which have two major isoforms known as the constitutive (cNOS) and the inducible (iNOS) isoforms, the latter mainly associated with injury-induced inflammation. cNOS localized in endothelial cells, is named eNOS, when localized in neurons is called nNOS (Moncada et al., 1997) iNOS, the isoform induced in macrophages and other cell types in response to endotoxin and/or various cytokines, has been found to be a major contributor to initiation and exacerbation of the CNS inflammatory/degenerative conditions. Activation of iNOS and NO generation become accepted as a marker and therapeutic target in neuroinflammatory conditions such as those observed in brain IRI (Pannu and Singh, 2006). Within 1 h of the cerebral IRI, eNOS is activated to produce a low concentration of NO considered as protective to brain vasculature (Chen et al., 2018). Huang et al. (1996), reported that in eNOS knockout mice cerebral IRI exacerbated the infarct volume compared with wild type mice, indicating a neuroprotective role of NO most probably by inducing vasodilatation and improving blood flow to penumbra. However, during the reperfusion stage, the activation of iNOS leads to the excessive production of NO, which generates reactive nitrogen species (RNSs) and causes exacerbation of cerebral IRI damage (Iadecola et al., 1995; Grandati et al., 1997). Zhao et al. (2000), found that knockdown of either iNOS or nNOS gene reduced the infarct volume in mice exposed to MCAO. Nash et al. (2018), demonstrated that nitrone 5, a NOS inhibitor, caused a reduction of infarct volume following permanent brain ischemia. Cumulatively these findings indicate that excessive production of NO is neurotoxic in cerebral IRI. The neurotoxic effect of NO is related to the reaction of NO to superoxide (O_2_^-^) generating peroxynitrite radicals (ONOO-), which amplify the IRI induced inflammatory process (Brea et al., 2009; Ding et al., 2014; Chen et al., 2018). In addition to its important contributions to inflammation, vasodilatation and oxidative processes, NO also plays a key role in the BBB disruption during cerebral IRI (Jiang et al., 2014).

#### Brain IRI and NF-Kappa B

NF-kappa B (NF-κB) is considered as a prototypical proinflammatory signaling pathway, largely based on the activation of NF-κB by proinflammatory cytokines such as IL-1 and TNF-α. However, gene knockout studies show that NF-κB proteins play both pro-inflammatory and anti-inflammatory roles (Lawrence, 2009). NF-κB, is a transcription factor composed of five subunits: RelA (p65), RelB, c-Rel, p105 (NF-κB1; a precursor of p50) and p100 (NF-κB2; a precursor of p52) (Ridder and Schwaninger, 2009) which bear nuclear localization sequence (NLS) and homo/heterodimerization motifs that allow them, upon translocation from the cytoplasm to the nucleus, to access and combines with κB sites in DNA promoter regions and drive transcription. NF-κB is a central regulator of inflammatory response in cerebral IRI (Harari and Liao, 2010). Among NF-κB subunits, p50 and p65 are the main inducers of inflammation and apoptotic cell death. In unstimulated cells, NF-κB is localized in the cytoplasm through binding to IκB (inhibitor of nuclear factor Kappa-B), which sterically blocks the function of NF-κB’s NLSs. After induction of cerebral IRI, the enhancing levels of expression of TLRs, IL-1, TNF-α, IL-1R, and TNF-R can stimulate phosphorylation of IκB through IκB kinase (IKK), release of NF-κB which translocate to the nucleus to activate its target genes (Chang and Huang, 2006). Knockdown of p50 leads to a smaller ischemic infarct in both transient and permanent cerebral ischemia models, as also observed in cerebral IRI mice expressing a conditional deletion of p65 (Inta et al., 2006). Compared with the sham group, the escape latency becomes shorter and the cerebral ischemic infarction volume was smaller when MCAO rats were treated with NF-κB inhibitor, PDTC. While MCAO rats were treated with NF-κB activator LPS, the escape latency became longer and the cerebral ischemic infarction volume was larger (Zhao et al., 2018). Postmortem human brain samples of IS patients further indicated increased levels of NF-κB p65 in the ischemic infarct area and necrotic core (Nurmi et al., 2004). A clinical study of 60 patients with acute IS found that the NF-κB is inhibited by the up regulation of transcribed IκBα mRNA and the impeded phosphorylation and degradation of IκBα is in correlation with improved clinical outcomes (Zhang F. et al., 2018). In addition, NF-κB also participate in inducing apoptosis and ameliorating BBB permeability in cerebral IR injury (Wang et al., 2010; Zhao et al., 2018). Because a significant body of data implying a protective role for NF-κB in stroke comes from deletions of specific NF-κB family members in mice with MCAO, it was proposed that the balance of NF-κB activation as neuroprotective or neurotoxic is mediated through diversity in homo-and heterodimer composition. Another variable that can impact the balance of NF-κB effect in stroke is time (Harari and Liao, 2010). The contribution of NF-κB activation in brain IRI now appears far from simple and many temporal and spatial variables could impact the balance of NF-κB effect toward neurotoxicity or neuroprotection.

#### TCM Neuroprotection in Cerebral IRI by Attenuation of the Inflammatory Response

Traditional Chinese medicine has been widely used in countries of south and east Asia patients with stroke or TBI for several centuries. In the last decade, many preclinical and clinical studies were focused on the therapeutic effects of TCM in cerebral IRI iduced by stroke or TBI. To discover and develop novel natural compounds, active ingredients, single herbs and combination formulas or prescriptions in TCM with therapeutic selectivity were investigated for the ability to confer neuroprotection in cerebral IRI diseases. A meta-analysis evaluating 28 trials that included 2780 stroke patients indicated that several Chinese herbal patented medicines were effective in recovery from stroke (Han et al., 2017). Similarly, a meta analysis systematic review of 14 high quality studies on animals with experimental TBI, indicated beneficial therapeutic effects of TCM, expressed by reduction of brain water content, improvement of BBB permeability, and decrease of TNF-α/NO expression (Yang et al., 2016). In many studies the beneficial neuroprotective effect of TCM was atributed to inhibition of inflammatory cytokines production and attenuation of the brain neuroinflammatory response. Some typical representative examples are provided in Tables 1–4.

**Table 1:** The neuroprotective effects of TCM herbs involves anti- inflammatory responses in IRI rodent models.

TCM herb	Rodent type	Anti-inflammatory target	Cerebral IRI-model	Reference
*Ilex pubescens*	Mice/rat	NO↓, IL-1β↓, TNF-α↓, TNOS↓, iNOS↓, cNOS↓ IL-10↑, NOS↓	BCCAO/MCAO	Fang et al., 2017; Yan et al., 2017
*Carthamus tinctorius L.*	Rat	IL-1β↓, TNF-α↓	BCCAO	Fu et al., 2016
*Gastrodia*	Rat	NO↓, iNOS↓, nNOS↓	MCAO	Zeng et al., 2006; He et al., 2016; Xian et al., 2016
*Dracocephalum moldavica* L.	Rat	TNF-α↓, IL-6↓, IL-8↓	MCAO	Jia J.X. et al., 2017
*Sophora japonica*	Rat	IL-1β↓	MCAO	Lao et al., 2005
*Ganoderma lucidum*	Rat	TNF-α↓, IL-8↓	MCAO	Zhang W. et al., 2014
*Panax notoginseng*	Mice	Trx-1↓, NF-κB↓, HSP70↓	MCAO	Zeng et al., 2014
*Acanthopanax polysaccharides*	Rat	IL-10↑, IL-1β↓, TNF-α↓	MCAO	Xie et al., 2015
*Rabdosia rubescens*	Rat	NO↓, NOS↓, IL-1β↓, TNF-α↓	MCAO	Miao et al., 2017
*Rhizoma Pinelliae Pedatisectae*	Rat	IL-1β↓, TNF-α↓	MCAO	Ye et al., 2016
*Kudiezi*	Patient	IL-6↓, IL-18↓, IL-10↑	IS	Liu X. et al., 2018

**Table 2:** The neuroprotection of active TCM components involves anti- inflammatory responses in IRI rodent models.

TCM-compound	Rodent type	Anti-inflammatory target	Cerebral IRI-model	Reference
*Ferulic acid*	Rat	ICAM-1↓, NF-κB↓, superoxide radicals↓, MPO↓	MCAO	Cheng et al., 2008
*Cholalic acid/hyodeoxycholalic acid*	Rat	IL-1β↓, TNF-α↓	MCAO	Hua et al., 2009
*Curcumin*	Rat	IL-1↓, TLR4↓, p-38↓, p-p38↓	MCAO	Huang et al., 2018
*Schisandrin B*	Rat	IL-1β↓, TNF-α↓	MCAO	Lee et al., 2012
*Puerarin*	Rat	TLR4↓, MyD88↓, NF-κB↓, TNF-α↓	MCAO	Zhou et al., 2014
*Matrine*	Rat	NF-κB p65↓, p53↓, c-Myc↓	MCAO	Xu et al., 2012
*Ampelopsin*	Rat	IL-1β↓, TNF-α↓	MCAO	Ye et al., 2017
*Icariside II*	Rat	IL-1β↓, TGF-β1↓, NF-κB↓, PPARα↑, PPARγ↑, IκB-α↓	MCAO	Deng et al., 2016; Xiong D. et al., 2016
*Tanshinone IIA*	Mice/gerbils	SOD↓, MDA↓, iNOS↓, TNF-α↓, NO↓, NF-κB↓, IL-4↑, IL-13↑	MCAO/BCCAO	Dong et al., 2009; Park et al., 2014
*Salvianolic acid B*	Mice	GFAP↓, Iba1↓, IL-1β↓, IL-6↓, TNF-α↓, Cleaved-caspase 3↓	MCAO	Fan et al., 2018
*Astragaloside IV*	Mice/rat	TLR4↓, MyD88↓, TRIF↓, TRAF6↓, NF-κB↓, NO↓, TNF-α↓IL-1β	BCCAO/MCAO	Li M. et al., 2017; Xu E. et al., 2017
*Salvianolic acid B and puerarin*	Rat	TNF-α↓, IL-1β↓, IL-6↓, TLR4↓, MyD88↓, NF-κB↓	MCAO	Ling et al., 2018
*Magnolol*	Mice	NO↓, TNF-α↓, NF-κB p65↓	MCAO	Liu et al., 2017
*Scutellarin*	Mice	IL-1β↓, TNF-α↓, iNOS↓, CSF↓, NF-κB↓	MCAO	Yuan et al., 2016; Wu et al., 2017
*Honokiol*	Mice	NO↓, TNF-α↓, RANTES/CCL5↓	BCCAO	Zhang et al., 2013
*Borneol*	Mice	iNOS↓, TNF-α↓, NO↓	MCAO	Chang et al., 2017
*Paeonol*	Rat	TLR2↓, TLR4↓, Iba1↓, NF-κB (p50)↓, IL-1β↓	MCAO	Liao et al., 2016
*Brazilein*	Mice	NOD2↓, TNF-α↓	BCCAO	Yan et al., 2016
*Tetramethylpyrazine*	Rat	TNF-α↓, IL-8↓, HMGB1↓, TLR4↓, NO↓, NOS↓	MCAO	Zhang C. et al., 2014, 2018; Chang et al., 2015
*MLC901*	Mice	IL-1β↓, IL-6↓, TNFα↓, TLR4↓	MCAO	Widmann et al., 2018
*β-Asaron*	Rat	IL-1β↓, TNF-α↓	MCAO	He et al., 2018

**Table 3:** The neuroprotective effects of TCM formula and decoction involves anti- inflammatory responses in IRI rodent models.

TCM	Rodent type	Anti-inflammatory target	Cerebral IRI- Model	Reference
*Gueichih-Fuling-Wan*	Rat	iNOS↓, COX-2↓, NO↓	BCCAO	Chen et al., 2016
*Herbal formula FBD*	Mice	PMNs↓, TNF-α↓, IL-1↓ IL-8↓, N F-κB↓	BCCAO	Lin et al., 2008
*Naoxintong*	Mice	LOX-1↓, pERK1/2↓, NF-κB↓	MCAO	Xue et al., 2016
*Shaoyao-Gancao*	Rat	IL-1β↓, TNF-α↓, MCP-1↓, IL-10↓, NeuN↑, IBA1↓, GFAP↓	MCAO	Zhang et al., 2016
*Danshen-Chuanxiong-Honghua*	Mice	IL-6↓, IL-1β↓, TNF-α↓	MCAO	Zhang et al., 2017
*Tao-Hong-Si-Wu-Tang*	Rat	HIF-1α↓, TNF-α↓, iNOS↓	MCAO	Wu et al., 2011
*Xueshuantong*	Rat	IL-1β↓, IL-17↓, IL-23p19↓, TNF-α↓, iNOS↓, Prx6↓, TLR4↓, p38↓, STAT3↑	MCAO	Wang X. et al., 2015
*Huangjiao granules*	Rat	IL-10↑, PI3K↑, p-AKT↑, p-mTOR↑, IL-1β↓, TNF-α↓	MCAO	Pan et al., 2017
*ZiYin HuoXue JieDu*	Rat	IL-1β↓, TNF-α↓, IL-8↓	MCAO	Chunyan et al., 2011
*Shengmai san*	Rat	TNF-α↓, NO↓	BCCAO	Li et al., 2013
*Yokukansan*	Gerbils	Iba1↓	BCCAO	Zhou et al., 2014
*Weinaokang*	Mice	MMP-9↓, p-ERK1/2↓, GRK2↓, NO↓, NOS↓	BCCAO	Zheng et al., 2010

**Table 4:** The neuroprotective effects of TCMs anti- inflammatory responses inTBI rodent models injury.

TCMs	Rodent type	Anti-inflammatory target	TBI- Model	Reference
*Artesunate*	Mice	*NF-kB*, IL-1β↓, TNF-α↓	CCI	Gugliandolo et al., 2018
*Tetramethylpyrazine*	Mice	IL-1β↓, TNF-α↓, iNOS↓	CCI	Wang et al., 2017
*Rhizoma drynariae*	Rat	IL-10↑, IL-6↓	CCI	Wang et al., 2016
*Modeifed Shengyu” decoction*	Rat	IL-1β↓, TNF-α↓, IL-10↑	CCI	Zhao et al., 2014
*MLC 601*	Rat	TNF-α↓	Fluid percussion injury	Tsai et al., 2015
*Longxuejie capsule*	Rat	TNF-α↓, NO↓	Weight-drop injury	Wang and Li, 2012

#### Herbs

(1) *Ilex pubescens* is an evergreen shrub mainly distributed in the south of China, which has been used to treat cardiovascular disorders, stroke and peripheral vascular disorders (Xiong H. et al., 2016). *Ilex pubescens* was found to reduce IRI in brain’s hippocampus and cortex by down regulation of NO and stimulation of Ca^2+^ dependent ATP enzyme activation (Yan et al., 2017). Fang et al. (2017), reported that *Ilex pubescens* can attenuate the cerebral IRI in MCAO rats. *Ilex pubescens* inhibited the production of NO due to inhibition of iNOS, cNOS and decreased production of IL-1β and TNF-α. However, *Ilex pubescens* therapy resulted with significant activation of IL-10 in brain tissue, sugestive of a neuroprotective effect toward cerebral IRI by affecting the balance of pro and anti-inflammatory cytokines.
(2) *Gastrodia elata* has been extensively used for therapy of cerebral IRI and other brain disorders for centuries in China. In MCAO models, *Gastrodia* therapy inhibited NO production, and decreased the activity of nNOS and iNOS (Zeng et al., 2006). In addition, *Gastrodia* protected the brain from BBB damage by inhibiting the expression of aquaporin-4 and enhancing the expression of tight junction proteins Occludin and Claudin-5 (He et al., 2016).
(3) *Kudiezi* injection was made of *Ixeris sonchifolia* (Bge.) Hance it was made using the whole herb as the injection used as the first line treatment of acute cerebral infarction. Liu and colleagues, enrolled a series of 67 patients with stroke, and divided them into Kudiezi group and control group. Compared with the control group, Kudiezi could improve the neurological deficits in IS patients on days 5 and 7 post-treatment, and the pro-inflammatory cytokine, IL-6 levels in serum was lower in Kudiezi group on days 3, 5, and 14, while the anti-inflammatory cytokine IL-10 levels were higher in Kudiezi group on days 5 and 14. In addition, the Kudiezi also decreased other pro-inflammatory cytokines such as IL-18 and matrix metalloproteinase-9 levels (Liu X. et al., 2018).
(4) *Carthamus tinctorius L.* (Fu et al., 2016), *Rhizoma Pinelliae Pedatisectae* (Ye et al., 2016), *Rabdosia rubescens* (Miao et al., 2017), and *Acanthopanax polysaccharides* (Xie et al., 2015) confered neuroprotection toward cerebral IRI by inhibiting the expression of pro-inflammatory cytokines, such as IL-1β and TNF-α. *Rabdosia rubescens* also suprressed the release of NO, reduced the activation of NOS, while *Acanthopanax polysaccharides* increased the release of anti-inflammatory ctyokines (IL-10). Both *Dracocephalum moldavica* (Jia J.X. et al., 2017) and *Ganoderma lucidum* (Zhang W. et al., 2014) confered neuroprotection by inhibiting production of pro-inflammatory cytokines, such as IL-8 and TNF-α. *Sophora japonica* (Lao et al., 2005) attenuated cerebral IRI by reducing the production of IL-1β, while *Panax notoginseng* (Zeng et al., 2014) confered neuroprotection by decreasing NF-κB activity, inhibiton of HSP70, and inhibition of SOD enzyme.

#### Active Components

(1) *Salvianolic Acid B*, extrated from the well-known Chinese herb named *Salvia miltiorrhiza*, has been used to treat patients with stroke from the ancient era (Li X.Y. et al., 2017). Ling et al. (2018), found that *Salvianolic Acid B* reduced ROS levels, inhibited apoptosis and enhanced mitochondrial membrane potential in the cobalt chloride treated PC12 cells (chemical ischemia), and attenuated the cerebral IRI in rat with MCAO in correlation to down regulation of the NF-κB and reduction of pro-inflammatory cytokine, such as TNF-α, IL-1β, IL-6. Fan et al. (2018), demonstrated that Salvianolic Acid B confered neuroprotection toward cerebral IRI injury by inhibition of astrocytes and microglia activation, leading to significantly down-regulation of the expression of TNF-α, IL-1β, and IL-6.
(2) *Tanshinone (TSA)*, another major compound of *Salvia miltiorrhiza*, was also characterized by its ability to protect the brain from transient IRI (Tang et al., 2014). *TSA* significantly reduced the ischemic infarct volume and improved neurological deficits via inhibition of ROS increase and suppression of NO production as well as reduction of the expression of NF-κB protein (Dong et al., 2009). Interestingly, 30 min pre-treatement of the gerbils with 10 mg/kg of *TSA* before induction of cerebral IRI, induced higher expression of anti-inflammatory cytokines (IL-4 and IL-13) compared with untreated brain IRI gerbils. However, the IRI area was not affected when IL-4 was injected into the brain lateral ventricle, indicating that pre-tretreamt with *TSA* may play a neuroprotective role in IR injury by affecting the balance of multiple cytokines (Park et al., 2014).
(3) *Honokiol*, is an isomer of *magnolol*. Both compounds are extracted from *Magnolia officinalis* and mainly used as an antifungal agents. *Honokiol* administered in a dose range of 0.7–70 g/kg, after cerebral IRI in MCAO mice, significantly inhibited the activation of NF-Kb-p65, and consequently reduced NO, TNF-α, and RANTES/CCL5 levels (Zhang et al., 2013). Liu et al. (2017), reported that magnolol treatement of MCAO rat reduced the brain infarct volume and water content, improved BBB function, and reversed the up regulation of TNF-α and IL-1β levels induced by OGD in brain microvascular endothelial cell cultures. Moreover, magnolol confered neuroprotection in corelation with increased level of expression of the tight junction proteins ZO-1 and occludin, indicating that the neuroprotective effect was mediated by anti-inflammatory effects and improval of BBB permeability.
(4) *Astragaloside IV* extracted from *Astragalusmembranaceus*, and widely used to treat the cardiovascular diseases in China, was recently found as neuroprotective on cerebral IRI model by anti-inflammatory, anti-oxidative and anti-apoptotic mechanisms. *Astragaloside IV* neuroprotective effects were mediated by TLR4/ MyD88 pathway and suppression of the expression of TLR4 and downstream adaptor proteins MyD88, TRIF, and inhibition of NF-κB. In addition, *Astragaloside IV* also significantly inhibited the expression and secretion of TNF-α and IL-1β (Li M. et al., 2017). Xu E. et al. (2017), reported that *Astragaloside IV* can improve the neurological deficit and reduce the cerebral ischemic infarction volume not only by inhibition of pro-inflammatory cytokines, but also by blocking the increase of ROS.
(5) *Scutellarin*, isolated from *Erigeron breviscapus*, has antioxidant and anti-apoptotic properties. In cerebral IRI, *Scutellarin* inhibited the activation of microglia and decreased the inflammatory response by blocking the release of IL-1β and TNF-α and activation of NF-κB signaling pathway. In addition, Scutellarin also reduced the NO production and corected the BBB permeability properties (Yuan et al., 2016; Wu et al., 2017).
(6) *Icariside II*, extracted from *Herba Epimedii*, is widely used for the treatment of cardiovascular disoders and amelioration of neurological deficts. Recent data indicated that MCAO mice treated with Icariside II (10 or 30 mg/kg, for 3 days) achieved a better neurological score and smaller infarct volume. The neuroprotective effect of *Icariside II* was mediated by the inhibition of inflammatory responses through NF-κB-p65/ IκB pathway (Xiong D. et al., 2016). Deng et al. (2016), further demonstrated that *Icariside II* reversed the activation of NF-κB-p65 by phosphorylation and degradation of inhibitory κB (IκB), which binds to NF-κB-p65 to inhibit the production of TGF-β and IL-1β. In addition, *Icariside II* also controled the inflammatory responses via peroxisome proliferator-activated receptor (PPAR) pathway by mediating the up regulation of PPARα and PPARγ. PPARs are a nuclear receptor family regulating inflammation, and activation of the PPAR signaling pathway can protect the cerebral damage, and therefore, PPAR agonists may be candidates for brain IRI therapy.
(7) *Tetramethylpyrazine*, is a famous compound extracted from *Ligusticum wallichii* (Chuan Xiong), used for treatment of neurovascular and cardiovascular disorders. In last two decades, evidence was accumulated to demonstrate that *Tetramethylpyrazine* plays a neuroprotective effect on cerebral IRI by different mechanisms including anti-inflammation, anti-apoptosis, anti-oxidant and attenuation of BBB disruption (Li L. et al., 2017; Xu S.H. et al., 2017; Zhang C. et al., 2018). *Tetramethylpyrazine* supressed the release of NO, reduced the activation of NOS through PI3K/Akt signaling pathway (Yan et al., 2015) and reduced the expression of pro-inflammatory cytokines, inluding IL-1β and TNF-α (Zhang C. et al., 2014), attenuated the activity of HMGB1, TLR4, Akt, and ERK (Chang et al., 2015).
(8) MLC901, extracted from nine Chinese traditional herbal components, could attenuate brain water content and cerebral infarction volume in mice with MCAO. MLC901 had the neuroprotective effect on the inflammation cascade through decreasing the pro-inflammatory cytokines, such as IL-1β, IL-6, and TNFα mRNA levels and activation of Toll-like receptor 4 (TLR4) signaling pathway (Widmann et al., 2018).

Many other active TCM components confered neuroprotection toward cerebral IRI by improving the neurological deficits, attenuation of the brain water content (edema), decrease of the infarct in temporal correlation with inhibition of the inflammatory response (Table 1). *Cholalic acid* and *Hyodeoxycholalic acid* (Hua et al., 2009), *β-asaron* (He et al., 2018), *Schisandrin B* (Lee et al., 2012), and *Ampelopsin* (Ye et al., 2017) were found to mitigate the release of IL-1β and TNF-α induced by MCAO in rats/mice. *Puerarin* (Zhou et al., 2014) and *Curcumin* (Huang et al., 2018) confered neuroprotection toward cerebral IRI injury by inhibition of the TLR4/MYD88 pathway, while *Puerarin* (Zhou et al., 2014), *Matrine* (Xu et al., 2012), and *Ferulic acid* (Cheng et al., 2008) inhibited NF-κB-p65 pathway in relation to their neuroprotective effect.

#### Formula and Decoction

(1) *Shaoyao-Gancao* decoction is an extract of the plants, *Paeonia lactiflora* and *Glycyrrhiza uralensi*s. This decoction confered neuroprotection toward cerebral IRI by inhibitng the production of pro-inflammatory cytokines and chemokines (MCP-1) in serum and brain tissue. *Shaoyao-Gancao* decoction significantly reduced the expression of IL-1β, TNF-α, MCP-1, and TGF-β1 in the brain tissue, and the expression IL-1β, MCP-1, and IL-10 in the serum (Zhang et al., 2016).
(2) *Danshen-Chuanxiong-Honghua* was found to improve neurological function, reduce cerebral ischemic infarct volume and brain edema in a dose-dependent manner. He also inhibited the expression of pro-inflammatory cytokines, including IL-1β, IL-6, and TNF-α. In additon, he inhibited cerebral IRI -induced apoptosis by reducing the protein level of Bax and increasing the protein level of Bcl-2 (Zhang et al., 2017).
(3) *ZiYin HuoXue JieDu* decoction is a traditional formula to treat patients with cerebral IRI. This neurotherapeutic effect was correlated with inhibition of the cerebral infarct volume, by suppressing the expression of inflammatory cytokines, such as IL-1β, IL-8, and TNF-α (Chunyan et al., 2011).
(4) *Xueshuantong* injection is a standardized product extracted from San-chi, which is the root of *Panax notoginsen*, a famous traditional Chinese herb in treatment of IS. Wang X. et al. (2015) investigated its neurotherapeutic effect in transient and permanent rat cerebral ischemia models and found significant increase in the rats’ body weight, decrease of the brain infarct region and brain swelling, inhibition of the mRNA expression of IL-1β, IL-17, and TNF-α via TLR4-p38 pathway. Moreover, *Xueshuantong*’ neuroprotection was correlated with downregulation of iNOS, and reduced levels of pro-inflammatory cytokines and ROS (Wang X. et al., 2015).
(5) *Huangjiao* granules therapy in rats with MCAO improved the neurological deficits and attenuated the cerebral ischemic infarct. The expression of pro-inflammatory cytokines, such as IL-1β and TNF-α, was inhibited in rats with IRI by *Huangjiao* granules upon intragastric administration. Whereas, the expression of anti-inflammatory cytokines, such as IL-10, was significantly up-regulated by *Huangjiao* granules, the expressions of PI_3_K, phosphorylated-AKT and phosphorylated-mTOR in the brain tissues also increased. Therefore, the proposed neuroprotective mechanism of *Huangjiao* granules therapy was described as activation of the PI_3_K/AKT/mTOR signaling pathway (Pan et al., 2017).
(6) *Weinaokang* and *Gueichih-Fuling-Wan* can alleviate cerebral IRI by reducing the NO production and attenuating the expression of iNOS (Zheng et al., 2010; Chen et al., 2016). It can significantly improve the neurological outcome infarction volume and brain water content in rats with brain IRI through inhibiting Erk1/2 phosphorylation and NF-κB activity (Xue et al., 2016) *Tao-Hong-Si-Wu-Tang* (Wu et al., 2011) conferred neuroprotection toward IRI by inhibition of both HIF-1α and TNF-α activity, and down regulation of the inflammatory response (decreased iNOS expression) and inhibition of apoptosis (inhibition of active caspase-3), while suppressed (Li et al., 2013) the expression of TNF-α and the production of NO (Table 3).

#### The Neuroprotective Effects of TCMs on TBI

(1) *Artesunate* is obtained from a Chinese plant *Artemisia annua*, used for centuries in China. *Artesunate* has been characterized as a multipotent agent with different pharmacological actions. Gugliandolo et al. (2018), used *Artesunate* (30 mg/kg) to treat TBI mice and found that *Artesunate* reduced the TBI-induced lesion and efficiently alleviated the neuroinflammation by inhibiting the expression of NF-κB and the proinflammatory cytokines, including IL-1β and TNF-α.
(2) *Tetramethylpyrazine* is a critical compound in *Ligustrazine*, and used as TCM analgesic drug. It was demonstrated that *Tetramethylpyrazine* had a neuroprotective effect on TBI as well as cerebral IRI. Wang et al. (2017) established a TBI mice model with CCI, and found that *Tetramethylpyrazine* significantly alleviated the periorbital hypersensitivity andthe neuroinflammation at the primary stage of TBI, by decreasing the levels of iNOS and the levels of the proinflammatory cytokines IL-6 and TNF-α.
(3) *Rhizoma drynariae*, a chinese herb, has the capabilty to modulate immune responses and has anti-inflammatory effects in mice with TBI. In a CCI rat model of TBI, *Rhizoma drynariae* reduced the brain lesion volume, corrected the neurologic deficits and cognitive function, attenuated anxiety-and depression-like behaviors in relation to decreased blood levels of IL-6 and increased IL-10 (Wang et al., 2016). In addition, *Rhizoma drynariae* increased the levels of IL-2 in plasma in a weight-drop rat model of TBI (Wang et al., 2012).
(4) Modified *Shengyu* decoction is improved based on *Shengyu* decoction. *Shengyu* decoction has been used to cure disorders with deficit in“qi”and “blood”induced by loss of blood for 100s of years. Zhao et al. (2014), used the modified *Shengyu* decoction at doses of 1.0 or 2.0 ml/200 g to treat rats with TBI, and the results demonstrated that modified Shengyu decoction treatment was neuroprotective, corrected the neurological deficits, brain water content and reduced the neuronal loss, in relation to enhanced expression of IL-10 and decreased expression of proinflammatory cytokines IL-1β and TNF-α.
(5) *MLC 601*, a TCM, was also used to treat TBI rats and found that *MLC 601* improved neurological motor deficits and reduced overexpression of TNF-α (Tsai et al., 2015). *Longxuejie* capsule alleivated the damage in a weight-drop rat model of TBI through reducing the level of NO and TNF-α in the serum (Wang and Li, 2012).

## Conclusion

Acute cerebral ischemia and TBI elicit an innate immune response that leads to a cascade of inflammatory events that culminates in necrotic death of neurons, glia and microcapilaries. Clinical and preclinical studies have found a direct relationship between elevated levels of inflammatory biomarkers and the risk for cerebral IRI. However, the signaling pathways that link these events are not fully understood. Central regulators of inflammatory response are NOSs and nuclear factor-kappa B (NF-κB). Their activation is required for the generation of the oxidative stress insult and for the transcriptional induction of many pro-inflammatory mediators involved in innate immunity, such as cellular adhesion molecules, cytokines, and growth factors. Therefore, neurotherapeutic modulation of these targets could potentially regulate the inflammatory processes in IS and TBI.

Traditional Chinese medicine has been recorded for more than 1000s of years in treatment of cardiovascular disorders and neurovascular disorders. Present review concisely presents several TCMs, including herbs, isolated active compounds from Chinese herbs and Chinese herbal formula and decoction, which exert neuroprotective effects on cerebral IRI and TBI, by suppressing at the molecular level the inflammatory cascade reactions associated with IRI pathological injury. It is proposed that TCMs rescue the injured neurons in the penumbra by shifting the balance of proinflammatory-anti-inflammatory cytokines toward neuroprotection. TCMs were also found to confer neuroprotection to the IRI brain toward inflammation-induced secondary injury in correlation to modulation of the expression of transcription factor-mediated proinflammatory genes. However, there are also several limits in the existing reports focusing on the neuroprotective effects of TCMs on inflammatory responses. First, majority of the reports only study one or two targets of the inflammatory pathway associated with cerebral IRI instead of investigating the multifactorial network of pathological pathways regulating neuroinflammation. Second, a large number of TCMs were found to exert a neuroprotective effect on brain IRI using rodent models, which differ greatly from patients. Novel clinical trials are required, to establish the TCMs neuroprotective effect in IS and TBI. Despite these limitations, the animal data has shown that TCM may be neuroprotective in the IS and TBI animal models. However, successful clinical translation of this neuroprotective strategy necessitates rigorous, robust, and detailed pre-clinical evaluation. Therefore, additional well-designed and well-reported experimental animal studies are needed which might be complemented with *in vitro* stroke/TBI-models.

## Author Contributions

WZ and LC designed the study. MF collected and analyzed the data. TP and YJ drafted the manuscript. TP, YJ, and PL did the critical revision of the manuscript.

## Conflict of Interest Statement

The authors declare that the research was conducted in the absence of any commercial or financial relationships that could be construed as a potential conflict of interest.

## Abbreviations

BBB: brain–blood barrier
BM: bone marrow
CCI: controlled cortical impact (TBI)
CNS: central nervous system
cNOS: constitutive nitric oxide synthase
CSF: cerebrospinal fluid
DAMP: danger associated molecular pattern protein
DD: death domain
eNOS: endothelial nitric oxide synthase
ERKs: extracellular regulated protein kinases
FADD: Fas-associated death domain
HMGB1: high mobility group box-1
HPA: hypothalamic-pituitary-adrenal axis
HSCs: hematopoietic stem/progenitor cells
HSP: heat-shock protein
ICAM: intercellular adhesion molecule
IgG: immunoglobulin G
IL: interleukin
iNOS: inducible nitric oxide synthase
IRI: ischemia- reperfusion- injury
IS: ischemic stroke
IκB: inhibitor of nuclear factor kappa-B
KEGG: kyoto encyclopedia of genes and genomes
LPS: lipopolysaccharide
MyD88: myeloid differentiation factor
MAPK: mitogen-activated protein kinase
MCA: middle cerebral artery
MCAO: middle cerebral artery occlusion
MCP-1: monocyte chemotactic protein-1
MMP: matrix metalloproteinase
MSC: mesenchymal
NF-kB: nuclear factor kappa B
NO: nitric oxide
NOS: nitric oxide synthase
OGD: oxygen and glucose deprivation
PC-PLC: phosphatidylcholine-specific phospholipase C
PDTC: pyrrolidine dithiocarbamate
PKC: protein kinase C
rtPA: recombinant tissue plasminogen activator
ROS: reactive oxygen species
SARM: sterile α and armadillo motif-containing protein
SNS: sympathetic nervous system
TBI: traumatic brain injury
TCM: traditional Chinese medicine
TGF-β: transforming growth factor-beta
TIRAP: TIR domain containing adaptor protein
TLR: toll-like receptor
TNF-α: tumor necrosis factor -α
TNFR1: Tumor necrosis factor receptor 1
TNFR2: tumor necrosis factor receptor 2
TSA: tanshinone
TRAM: TRIF-related adaptor molecule
VCAM: vascular cell adhesion molecule.
